# A dropout-regularized classifier development approach optimized for precision medicine test discovery from omics data

**DOI:** 10.1186/s12859-019-2922-2

**Published:** 2019-06-13

**Authors:** Joanna Roder, Carlos Oliveira, Lelia Net, Maxim Tsypin, Benjamin Linstid, Heinrich Roder

**Affiliations:** Biodesix Inc, 2970 Wilderness Pl, Ste100, Boulder, CO 80301 USA

**Keywords:** Machine Learning, Molecular diagnostics, Regularization, Boosting, Ensemble average

## Abstract

**Background:**

Modern genomic and proteomic profiling methods produce large amounts of data from tissue and blood-based samples that are of potential utility for improving patient care. However, the design of precision medicine tests for unmet clinical needs from this information in the small cohorts available for test discovery remains a challenging task. Obtaining reliable performance assessments at the earliest stages of test development can also be problematic. We describe a novel approach to classifier development designed to create clinically useful tests together with reliable estimates of their performance. The method incorporates elements of traditional and modern machine learning to facilitate the use of cohorts where the number of samples is less than the number of measured patient attributes. It is based on a hierarchy of classification and information abstraction and combines boosting, bagging, and strong dropout regularization.

**Results:**

We apply this dropout-regularized combination approach to two clinical problems in oncology using mRNA expression and associated clinical data and compare performance with other methods of classifier generation, including Random Forest. Performance of the new method is similar to or better than the Random Forest in the two classification tasks used for comparison. The dropout-regularized combination method also generates an effective classifier in a classification task with a known confounding variable. Most importantly, it provides a reliable estimate of test performance from a relatively small development set of samples.

**Conclusions:**

The flexible dropout-regularized combination approach is able to produce tests tailored to particular clinical questions and mitigate known confounding effects. It allows the design of molecular diagnostic tests addressing particular clinical questions together with reliable assessment of whether test performance is likely to be fit-for-purpose in independent validation at the earliest stages of development.

**Electronic supplementary material:**

The online version of this article (10.1186/s12859-019-2922-2) contains supplementary material, which is available to authorized users.

## Background

Lack of success in developing adequately validated, clinically useful molecular diagnostic tests remains a major hurdle in providing precision medicine to patients [1]. In addition to technical issues associated with lack of standardization and reproducibility of some technologies [1–4], there is often a lack of sample sets with adequate, well curated clinical data available for test development. Prospective studies designed to collect specimens from large cohorts of subjects in which the test is intended to be used are expensive and hard to justify when probability of successful test generation may be low. Hence, it is often necessary, at least in a feasibility or pilot stage, to make use of retrospectively collected sample sets. These sets may be pooled from different sources and not from the intended use indication of the test. Use of such “convenience sample sets” can lead to bias or confounding of the clinical question being studied; this can result in either failure to make a possible discovery or false positive test discovery. Working with suboptimal discovery sample sets and limited associated clinical data can also cause development of tests that are poorly suited to address real world clinical questions.

Even when appropriate test development cohorts are available, statistical hurdles may remain [5, 6]. Often there are more attributes measured per sample, *p*, than there are samples, *N*; while *p* may be of the order of thousands or tens of thousands, *N* is frequently only a few hundred, or even lower. This high-dimensional data regime presents statistical challenges [7, 8] and necessitates the use of good data analytical practices to try to minimize overfitting of the classifier to incidental details of the sample set [5, 6]. These difficulties combine to make false test discoveries more common than successful introductions of precision medicine tests into real world clinical settings.

**Fig. 1:**
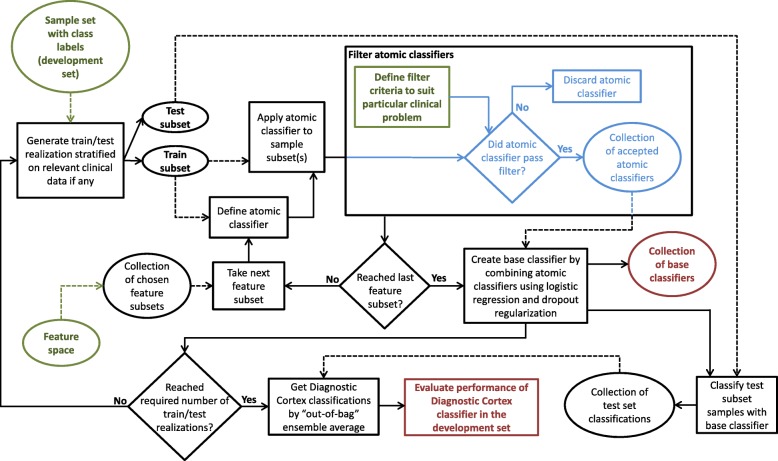
Classifier development architecture for dropout-regularized combination approach

We propose a novel approach optimized for development of precision medicine test discovery. It addresses some of these data analytical issues and allows better tuning of test development towards real clinical needs. The method incorporates concepts from traditional machine learning and recent advances in deep learning [9, 10] and it is hierarchical in structure. A flowchart of the approach is shown in Fig. 1. Many simple “atomic classifiers” are constructed with each using a small subset of the *p* attributes or features. These atomic (here k-nearest neighbor (kNN) [11]) classifiers are applied to the training set and filtered so that those failing to demonstrate even a minimal level of classification power are discarded. The filtered atomic classifiers are combined using logistic regression with strong regularization using a dropout approach to minimize overfitting. This process is repeated for many random splits of the development sample set into training and test sets. The continuous variable outputs of each of these multiple classifiers are ensemble averaged (“bagged” [12]). Finally, a binary classification can be obtained by application of a threshold selected during test development.

This dropout-regularized combination (DRC) classifier development approach was specifically designed to work well in the *p* > *N* (or *p* > > *N*) case, while minimizing the potential for overfitting and promoting the ability of the developed tests to generalize to unseen datasets. Further, use of “out-of-bag” estimates [13] across the ensemble average makes it possible to obtain accurate performance estimates for these tests from relatively small development sets. Reliable development set evaluations can reduce false discoveries and allow a robust preliminary assessment of whether a test has adequate performance for clinical utility. The method facilitates the design of clinically relevant tests through its capability to filter atomic classifiers. Discarding atomic classifiers which show no utility for the classification task enables both tuning of test performance and addressing any known confounding factors and bias that may be present in development cohorts. Any kind of expression data can be used as the basis for classification, and multiple kinds of clinical data (e.g., categorical, continuous, censored time-to-event) can be incorporated in the classifier development process. While the DRC approach has been used primarily with protein expression data in settings based on time-to-event data [14–16], it can be used with expression data from any reproducible source (e.g., proteomic and/or genomic). Here, we concentrate on its application to gene expression mRNA datasets in binary classification problems.

The goals of this study were:

- to assess the ability of DRC classifiers to generalize to unseen datasets as a function of number of samples available for development;
- to compare the performance of DRC classifiers with the performance of classifiers created using related approaches and a field standard, Random Forest (RF) [17, 18]; and
- to demonstrate the ability of the DRC method to deal with classification tasks plagued by known confounders.

To these ends, we selected several mRNA datasets from the Gene Expression Omnibus (GEO) database suitable for classifier development tasks in the precision medicine setting. We required:

- that the datasets have sufficient associated clinical data to formulate a meaningful classification task; and
- the existence of two independent sets, so that one could be used for development and the other could be set aside for independent validation.

To assess the ability of our approach to create high performing classifiers with accurate performance estimates from small sample sizes we selected two datasets collected from patients with prostate cancer and aimed to differentiate patients surviving 10 years or more after sample collection from those dying within the 10-year period. Datasets collected to investigate post-surgery prognosis for non-metastatic non-small cell lung cancer (NSCLC) patients were chosen for our assessment of the classifier development methodology’s ability to deal with known confounders. Here the clinical question chosen for investigation was the prediction of four-year survival post-surgery. Full details of the datasets and classifier development methods and parameters are provided in the Methods section.

## Results

### Ten-year survival for prostate cancer: testing the ability of the classifier development method to work well with small datasets

The classification task was to differentiate patients with prostate cancer still alive after 10 years of follow up from those dying within the 10-year period. mRNA expression data for 343 genes (features) were available for a development cohort (GSE16560) and a validation cohort (GSE10645). A description of the patient cohorts is given in the Methods. The atomic kNN classifiers (k = 7) were constructed using individual features and pairs of features. Only atomic classifiers demonstrating a minimal level of classification power were combined in the dropout regularized logistic regression. Specifically, when applied to their training set, the atomic classifiers had to achieve a classification accuracy greater than 0.68. Parameters defining the DRC approach were held fixed throughout this investigation with no tuning to improve performance. Values of all classifier parameters are provided in the Additional file 1.

First, the classification characteristics of the development and validation cohorts were compared. Nine randomly selected realizations of 168 patients (84 alive at 10 years and 84 dying before 10 years) were drawn from the GSE16560 cohort of 261 patients. A classifier was generated from each of these nine development set realizations using the DRC approach. Classifications of the development cohort were generated by out-of-bag estimate for each classifier and each classifier was also applied to the validation cohort. Receiver-operating characteristic (ROC) curves were constructed by varying the choice of threshold for creating the binary classification from the continuous variable test output. The average area under the ROC curve (AUC) across the 9 classifiers generated for the 9 development subset realizations was 0.634 (standard error (SE) = 0.010) for the development sets and 0.722 (SE = 0.008) for the validation cohort. Then the development and validation cohorts were reversed, so that classifiers were developed on the 9 subsets of 168 samples (84 in each class) randomly drawn from the validation cohort. Out-of-bag classifications were generated for the validation cohort and the 9 classifiers were applied to the development cohort. The resulting average AUC was 0.787 (SE = 0.014) for the 9 classifiers developed on the validation cohort, with an AUC of 0.658 (SE = 0.003) on the whole development cohort. Comparison of these two results indicated that the validation cohort (GSE10645) was substantially easier to classify than the development cohort (GSE16560). The latter was used to provide the development sets in the following studies exploring the dependence of classifier performance on development set sample size.

**Fig. 2:**
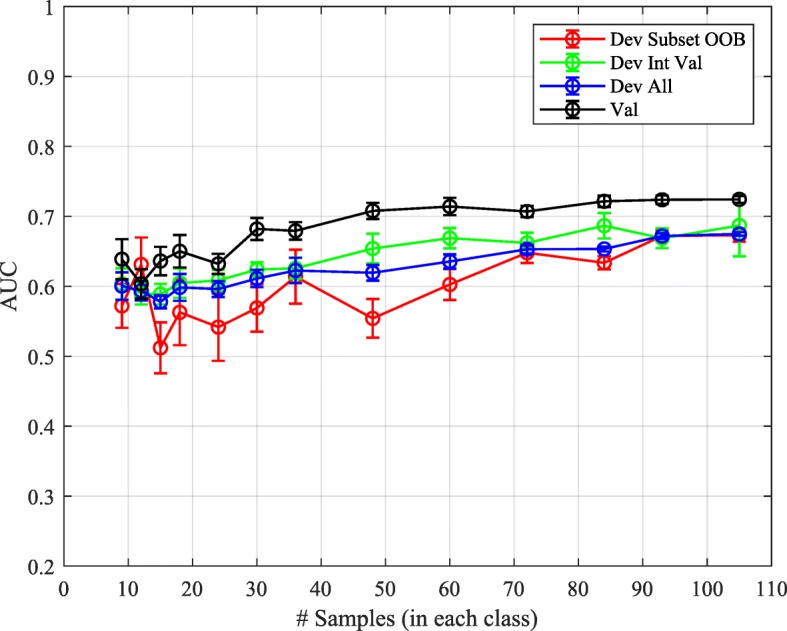
AUC averaged over 9 development subset realizations for DRC classifiers developed for subsets of size 210, 186, 168, 144, 120, 86, 72, 60, 48, 36, 30, 24, and 18 evaluated for the development subset by out-of-bag estimate (Dev Subset OOB), for development set samples not used for training (Dev Int Val), for all development set samples (Dev All), and for the independent validation set (Val)

Developing on 9 randomly selected subsets of the development cohort with subset sizes varying from 105 to 9 per class yielded the results shown in Fig. 2. Classifier performance on the development cohort was assessed using out-of-bag estimators on the development subsets, internal validation on the samples not used in each development subset, and across the whole development cohort (combining out-of-bag results for the development subset samples and standard classification for the internal validation samples). Similar results were obtained for all assessment methods. However, there was a general tendency that out-of-bag estimators slightly under-estimated classification performance for the smallest subset sizes. Performance assessments from the very small sample sizes were highly variable. This could be at least partially due to the inability of the smallest training sets to represent adequately the population to be classified.

Performance did not decrease much with decreasing subset size for development subsets with at least 100 patients (50 per class). Below this point there was some decrease in performance, but residual classification power in validation was maintained even for the smallest set with only 9 patients per class. Importantly, the difference between performance on the development subsets and the independent validation cohort remained similar regardless of the development subset size. Hence, our methodology generated non-inflated and generally reliable performance estimates for good performing classifiers down to very small development set sizes.

**Fig. 3:**
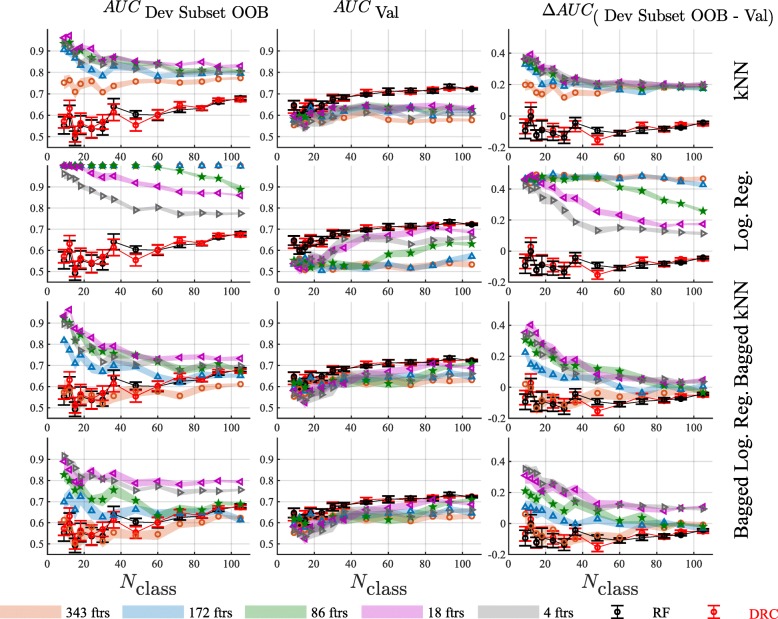
Results are shown for a single kNN classifier (1st row), a single logistic regression classifier (2nd row), bagged kNN classifiers (3rd row), and bagged logistic regression classifiers (4th row) as a function of the development subset size, for all 343 features, and 172, 86, 18, and 4 features, as selected by t-test *p*-value on the development subset. Left panels show average AUC on the development subset, center panels show average AUC on the validation set and right panels show the difference in AUC between the development subset and the validation set. Results for classifiers made with DRC and RF are also shown in each figure for comparison. Development subset AUCs are assessed within subset by out-of-bag estimates. Error bars show the standard error of the averages for DRC and RF and the colored bands show the standard error of the averages for the alternative classification methods

These results for the DRC method were compared with five other classifier development approaches, see Fig. 3: Random Forest (all rows), simple kNN (k = 7) (1st row), simple logistic regression (2nd row), bagged kNN (k = 7) (3rd row), and bagged logistic regression (4th row). Each plot shows the results using all available 343 features, 172 features, 86 features, 18 features, and 4 features, with feature selection by t-test for the latter 4 classifier development methods. The parameters used for each classification method are specified in the Additional file 1. No feature selection was necessary for DRC or RF. Figure 3 illustrates that, on the independent validation cohort (Fig. 3 center panels), classifiers developed using DRC or RF performed very similarly and uniformly as well as, or considerably better than, the other methods, even allowing for reduction in the number of features used for the alternative methods.

For single kNN, performance was very poor when all features are used, as expected [5]. Performance improved with feature selection, but did not approach the level of the DRC classifiers. Performance in validation decreased with reduction in sample size in a similar manner to that of DRC and RF, but smaller development subset sizes led to drastic increases in performance estimates from the development subset. This effect was mirrored by the persistent overestimation of performance, which increased dramatically as development subset size decreased (Fig. 3, first row leftmost panel). For logistic regression, performance in validation was better for small numbers of features than it was for kNN with any number of features for large development subset sizes. However, it still did not exceed that of the DRC or RF classifiers. Performance deteriorated dramatically for development subset sizes below 50 per class. Use of more than a few selected features or sample sizes less than 50 per class led to extremely overoptimistic performance estimates from the development subset, as shown in the rightmost panel of the second row of Fig. 3. To test whether these differences were due to the bagging component of DRC or RF, we also investigated bagged versions of kNN and logistic regression (3rd and 4th rows of panels of Fig. 3).

Bagging improved performance in validation for both methods quite substantially over the non-bagged classifiers, though still not surpassing that of DRC or RF. It did not, however, dramatically reduce the overestimation of performance from the development subsets, except for the cases where all features were used. In these cases for both bagged kNN and bagged logistic regression, as shown in the rightmost panels of the third and fourth rows of Fig. 3, performance estimates from the development subsets did not overestimate performance in the validation set. However, here overall performance in validation was extremely low anyway (center panels of third and fourth rows of Fig. 3).

Bagging alone was not sufficient to bring performance to the level of the DRC or RF classifiers or to provide much improvement in the accuracy of development set performance estimates. Not surprisingly, regularization was key to attaining better performance in the setting of small sample sizes and relatively many features. For this problem, the use of dropout regularization with a logistic regression combination performed as well as the standard RF model, which regularizes through its random selections of features used per tree node.

### Ten-year survival for prostate cancer: testing the ability of DRC and RF to work well for a dataset with very many, but few useful, features

**Fig. 4:**
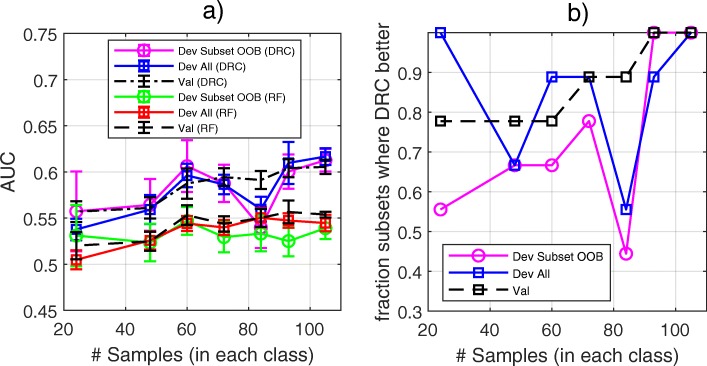
**a** AUC averaged over development subset realizations as assessed for the development set via within subset out-of-bag estimates (Dev Subset OOB) and for the independent validation set (Val). Error bars show standard error. **b** Proportion of development subset realizations with larger AUC for DRC than for RF as a function of development subset size for out-of-bag assessment within development subset (Dev Subset OOB), whole development set (OOB for samples used in training) and for the independent validation set (Val)

The prostate cancer dataset used for the first investigation was augmented by the addition of 10,000 randomly generated features to simulate the situation of a typical molecular dataset size with a small proportion of features useful for the desired classification task. DRC and RF classifiers were constructed for 9 randomly selected subsets of 105, 93, 84, 72, 60, 48, and 24 samples per class each to explore the ability of each method to classify based on small sample sets with very many, but few useful features. The parameters were kept the same as above, except that for the DRC approach the atomic classifiers created were restricted to those using single features and only pairs of features for which both of the single feature atomic classifiers passed filtering. Results are shown in Fig. 4. Even for the largest sample subset size, *N* = 105 in each class, the RF classifier showed very limited performance. The average AUC across subset realizations was 0.539, 0.545, and 0.554 for the development out-of-bag estimate, the whole development set (with samples used in training classified out-of-bag) and the independent validation cohort, respectively. The performance of the DRC classifier was systematically better than RF, with the DRC showing higher AUC for the majority of subset classifiers in independent validation, Fig. 4b. It is noteworthy that here the performance is similar in validation and development, so that the inclusion of very many additional noisy features has led to the generation of classifiers that no longer have better performance on the validation cohort than on the development set.

This investigation illustrates how the DRC method, with the filtering step, allows for a more efficient extraction of the small amount of useful information from the large amount of noise than is possible with a standard RF methodology. When only a very small fraction of features contains useful information, most trees in the RF will not access enough useful features to achieve any reasonable classification power. This issue does not arise in the dropout-regularized method, as all features can be used with each training/test set realization, and most of the large number of features with no information can be discarded during the filtering process. Features which, by random chance, are useful for classification within the training set for the ensemble realization are maintained, and these will still impact the performance of the final classifier. However, the features that passed filtering to be used for classification in the situation without additional noisy features also pass the filtering with the addition of noisy features. Provided that these informative features are not overwhelmed by the noisy features which incidentally pass filtering, construction of a classifier with utility is possible. The performance may be somewhat diminished, however.

### Four-year survival for NSCLC cancer: testing the ability of the classifier development method to deal with confounding effects

The classification task was to identify patients with non-small cell lung cancer (NSCLC) who lived longer than 4 years or died within 4 years after surgery. This was investigated with two datasets with 15,005 genes in common. Details of the sample cohorts used and classification parameters are given in the Methods and Additional file 1.

First, we investigated the association between gene expression and histology. Using mRNA expression data from 179 patients (43 squamous cell histology, 136 non-squamous histology) from the GSE50081 data set, a DRC classifier was constructed to differentiate squamous from non-squamous histology. Determination of histology from mRNA expression is a relatively easy classification problem and the classifier achieved an AUC of 0.870, with AUC = 0.896 in an independent validation cohort (GSE42127, *N* = 144 (33 squamous, 111 non-squamous)). This level of accuracy indicates that histology has the potential to be a strong confounding factor in developing other tests based on mRNA expression data from tissue from NSCLC patients.

We then designed a toy problem using these datasets to illustrate how known confounding factors can be dealt with by the filtering in the DRC approach. We set the goal of differentiating subjects with NSCLC surviving at least 4 years post-surgery from those dying before 4 years. A development subset was selected from the GSE50081 dataset by taking 35 subjects who survived longer than 4 years (28/7 squamous/non-squamous cell tumors) and 53 subjects who survived less than 4 years (12/41 squamous/non-squamous cell tumors). The problem is therefore constructed to be strongly confounded by tumor histology. The independent validation cohort (GSE42127, 72 surviving longer than 4 years, 33 dying within 4 years) represented a realistic, unconfounded, post-surgery NSCLC population of patients with tumors of squamous and non-squamous histology having survival outcomes less than and greater than 4 years.

**Fig. 5:**
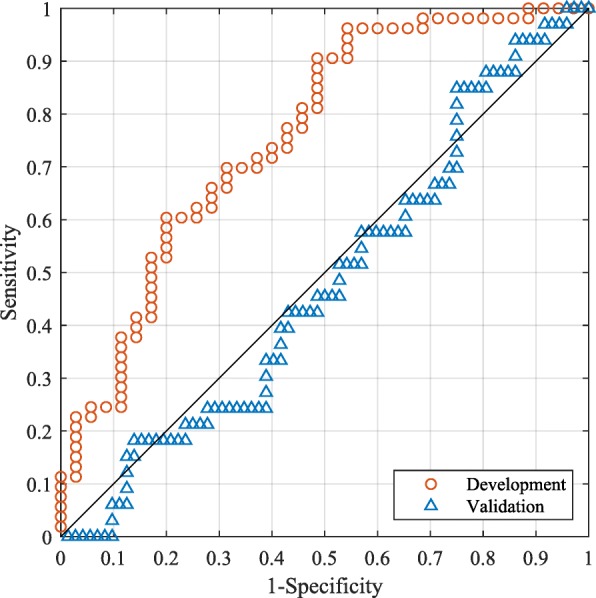
Results are shown for the classifier trained on the problem confounded by tumor histology for differentiation of subjects with NSCLC surviving at least four years post-surgery from those dying before four years. The ROC curves correspond to the case when no additional filtering constraint is applied using data from patients with non-squamous histology with insufficient follow up

Using the DRC method, a classifier was constructed with the confounded subset of 88 samples (35 alive at 4 years) to differentiate patients who survived longer than 4 years from those who did not. First, simple filtering was used with atomic classifiers retained in the regularized combination if they demonstrated a minimal ability to classify their training set accurately. The exact filtering used and other parameters are specified in the Additional file 1. As expected and illustrated in the ROC curves of Fig. 5, the classifier performed well when assessed on the development subset, but failed dramatically on the independent validation cohort. The classifier we constructed largely classified patients based on their tumor histology rather than their survival outcome. (Within the validation set, 18 samples out of the 28 (64%) classified as Alive at 4 years had squamous histology, while only 7 out of the 77 classified as Dead at 4 years (9%) had squamous histology.)

**Fig. 6:**
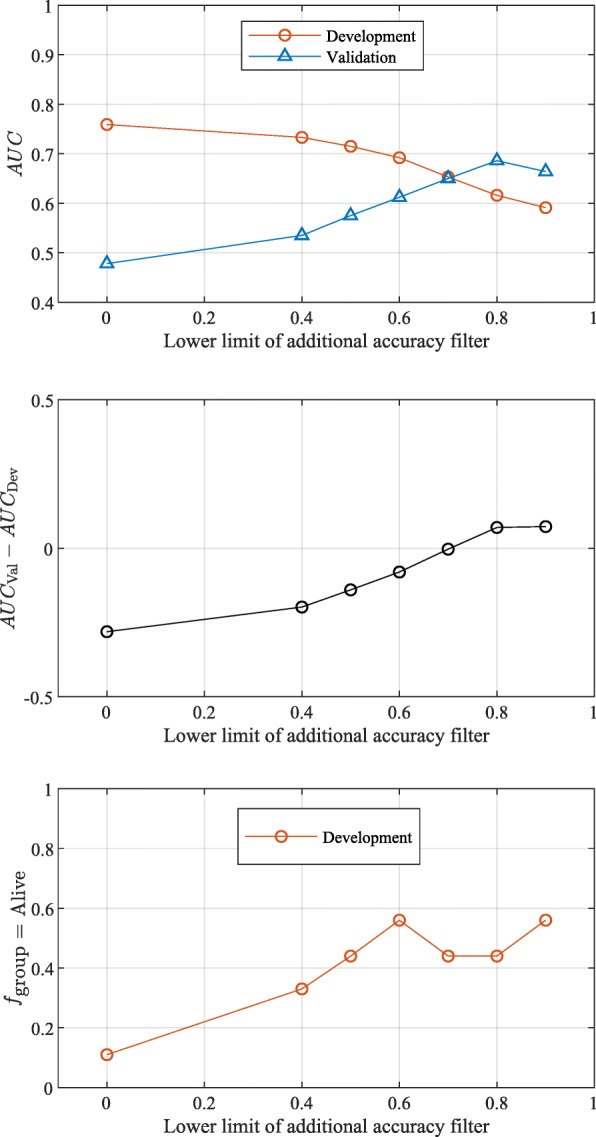
Performance for differentiation of subjects with NSCLC surviving at least four years post-surgery from those dying before four years is shown as a function of the lower accuracy limit of the additional filtering constraint applied using patients with non-squamous histology with insufficient follow up. First panel: AUC for the development subset and validation set; second panel: difference in AUC between development subset and validation set; third panel: fraction of the 9 subjects with insufficient follow up set aside for testing classified as Alive. The upper accuracy limit of the additional filtering constraint was set to 1.0 in all cases

To overcome this confounding effect, the filtering step used within the DRC approach was adjusted. In the GSE50081 dataset, there were nine patients with non-squamous histology with insufficient follow up to be unambiguously classified as alive or not at 4 years. Data from these samples, which could not be used for training due to this insufficient follow up, were used as an external filtering set. In addition to the simple metric of a minimal level of classification accuracy on the training set (used above), we now required that patients in the external filtering set should not all be classified as dying before 4 years. The results are shown in Fig. 6 for different levels of filtering on the external filtering set (i.e., threshold for the proportion of patients classified as Alive). Although the AUC of the development subset (first panel) decreased as the additional filtering on the external filtering set was tightened, the performance of the classifier in the validation cohort improved. The fraction of patients in the external filtering set that were classified as Alive at 4 years is shown in the third panel as a function of the lower limit of the additional filtering constraint: when it saturated (for lower limits of the additional filtering higher than about 0.6), the performance estimates in the development subset and in the validation cohort were close to each other, with no systematic overestimation of true classifier performance from the development set. The convergence behavior of the performance of the classifier on the external filtering set could, thus, be used as a criterion for deciding the optimal level of additional filtering. The additional filtering constraint allowed us to progressively (as the additional filtering was tightened) select a bigger fraction of the total number of atomic classifiers used in the logistic regression step that was able to distinguish between subjects with NSCLC surviving at least 4 years post-surgery from those dying before 4 years without using tumor histology as a surrogate.

This illustrates how the filtering component of the DRC approach can be used with an external filtering set of samples, either from a separate cohort or carved out of the development set, to monitor and deal with the effect of known confounders in the available samples.

## Discussion

The results presented here show the potential of our dropout regularized combination classifier development approach for tackling problems in the *p > N* and *p > > N* regime. The incorporation of the concepts of bagging, boosting, and regularization into the hierarchical structure allow the creation of classifiers tuned to specific clinical problems using the kinds of sample sets available, with the advantage of reliable performance estimates from the development set. This gives researchers not only the ability to design tests appropriate to specific clinical applications, but also increased confidence that classifiers promising performance adequate for clinical utility in development will reproduce this in validation. This approach has already been used as outlined here to design new tests for detection of hepatocellular carcinoma in high risk patients [19].

Many clinical problems do not lend themselves to a simple classification into two groups measured by sensitivity and specificity or accuracy. For example, it is often of interest to identify patients with better or worse prognosis on a particular treatment regimen, or patients who benefit most from one therapy relative to another. Choice of the metric for filtering of atomic classifiers can be made to tune test development to a particular clinical goal, e.g. better survival or better progression-free interval. It is easy to construct metrics using all kinds of clinical outcome data, including categorical (such as radiological response), continuous (such as change in body mass) or time-to-event data with censoring (such as overall or progression-free survival). One example where these classifier development methods have been applied to a problem involving endpoints other than binary is identification of patients with advanced melanoma who are likely to have better or worse outcomes following immunotherapy [14, 15]. These studies also incorporated an extension that allows a simultaneous refinement of classifier and training classes in a semi-supervised approach which is particularly useful for problems in which training class definitions are not a priori obvious.

While the examples included here demonstrate some advantages of the DRC approach, they also show that performance for some problems is similar to that of the Random Forest. It is possible that some tasks may be better treated with our approach, while others are better treated with a tree-based approach such as Random Forest. The way in which the data is processed is distinctly different between the two methods: DRC has a data abstraction via the atomic kNN classifiers, utilizes information from highly correlated features differently, emphasizes incorporation of all features with even minimal utility, and the logistic regression tends to favour consistency across atomic classifier classification outputs; RF selects the optimal feature at each node via the CART approach and may have advantages in combining features with more orthogonal information. The relative utility of these approaches may therefore depend on the particular problem investigated and the setting in which the developed test is to be used. A large-scale benchmarking study, similar to that comparing logistic regression with RF in problems with *p < N* [20], would be useful to try to elucidate which classes of problem might be better suited to which approach in this *p > N* regime. In the context of development of tests for precision medicine, it would be important to add to the classification performance criteria used in Couronné et al. [20], an assessment of the ability of the test to generalize to an unseen but similar population and some measures of reproducibility of test classification to repeat measurements of the molecular data. These latter two considerations are key metrics for real-world molecular diagnostics.

The novelty of our approach lies in the combination of the machine learning techniques used and the main goal is consistently creating tests with reliable associated performance estimates tuned to particular clinical problems rather than optimal levels of performance. While we believe that the bagging, boosting, and strong regularization are elements essential to the ability of the approach to meet these goals, the particular way that these elements are implemented is likely not so crucial. Investigation of variants using other kinds of atomic classifiers and alternative methods of atomic classifier combination and regularization are underway. It would also be possible to add elements of our approach, such as within-bag filtering, to Random Forest-based classification schemes. Extensions of our approach which allow the incorporation of binary features or features with a small number of categorical values are also ongoing. These possibilities would increase the ability of the DRC approach to use data optimally from one or more sources, such as those now available from multi-omic patient characterization.

It should be noted that while this approach tackles some of the bioinformatics challenges inherent in the development of molecular diagnostic tests, many other hurdles to establishing a novel test in clinical practice still exist. Not least of these is the need for a personalized medicine test to work using data obtained from a measurement platform on a sample type that is practical for use in a real-world setting, high-throughput and reproducible. Transfer of signatures discovered using data gathered using a technology that cannot be applied in the clinic to other more-easily utilized measurement platforms is a key point of failure in the traditional approach to molecular diagnostic test development. The design of tests with the DRC classifier development approach using data collected with well-standardized methods suitable for direct transfer into clinical practice could allow a rapid and reliable assessment of whether resulting tests can perform well enough to have utility in everyday clinical practice. Prospective validation of any resulting test in the clinic is of course still necessary to unequivocally establish its practical clinical utility.

## Conclusions

The dropout-regularized combination method is a flexible approach to classifier development, well-suited to situations in which sample sets are small and have more attributes than instances. Its hierarchical structure, which incorporates bagging, boosting, and dropout regularization, allows for mitigation of known confounding factors and tuning of the classifiers towards performance goals. The DRC approach allows the design of molecular diagnostic tests addressing particular clinical questions together with reliable assessment of whether test performance is likely to be fit-for-purpose in independent validation at the earliest stages of development.

## Methods

### Classifier development methods

#### Dropout regularized combination (DRC) classifiers

The overall structure is illustrated schematically in Fig. 1. The set of patients available for development is randomly split into training and test sets (“training/test set realizations”) many times. An ensemble average (“bagging” [12]) over these training/test split realizations allows every sample in the development set to contribute to the performance estimate of the final classifier via an “out-of-bag” estimate [13], i.e. the classification for a given sample in the development set is evaluated only over the subset of realizations where the sample is in the test set and not in the training set. This allows for more reliable and generalizable classifier performance estimates to be generated from the development set alone. Each training/test set realization is generated stratified by class to yield equal numbers of samples in each training class. This is necessary to avoid bias in the subsequent logistic regression.

For each training/test split realization, many classifiers (“atomic classifiers”) are built using subsets of the features from the feature space. The exact method of exploring the multitude of possible atomic classifiers is not important, as long as the sampling has adequate diversity. Typically we construct all possible combinations of a small number of features, such as all singlets, pairs, and triplets of features. Here we use k-nearest neighbor (kNN) classifiers [11] with fixed k for atomic classifiers, but any other methods that produce a classifier from a number of features and class-labelled instances could be used. Each atomic classifier is applied to its training set and/or some independent sample set and the resulting classification groups are used to evaluate a metric appropriate for the particular classification problem. The atomic classifiers are filtered so that only classifiers demonstrating some minimal level of performance based on the chosen metric pass filtering and are used further in the process. This approach uses the principle of boosting [21] – that many classifiers of decent performance can be combined into an overall classifier with at least as good, or better, performance.

Once the atomic classifiers have been filtered and poorly performing classifiers eliminated, the remaining atomic classifiers are combined to create one base classifier per training/test split realization. Our studies have used logistic regression over the training set samples for this purpose. As there are very many atomic classifiers that pass filtering, strong regularization is essential to avoid overfitting. We used the concept of dropout, a common regularization technique used in the training of deep learning nets [22]. Dropout can be thought of as a way of adding noise to a system which thus minimizes the likelihood of overfitting to training data. The application of dropout to logistic regression has been studied and shown to be first-order equivalent to an L_2_ regularizer [23, 24]. Our regularization method is implemented as follows: From the pool of atomic classifiers passing filtering, we randomly select a small number of atomic classifiers, *m*, smaller than the number of samples in the training set and typically 10 or less. We perform the logistic regression to calculate weights for combining this subset of atomic classifiers. We repeat this many times, enough so that each atomic classifier is drawn many times. The weight for each atomic classifier is averaged over many dropout iterations to give the weights for the final logistic combination.

The final level of the hierarchy is an ensemble average of the base classifiers (bagging over the training/test split realizations [12]). This can be carried out as a majority vote of binary outputs after application of a threshold to the logistic function output or as an average over the continuous output of the logistic function followed by application of a threshold to the average. In these studies we use the latter approach to ensemble average over the logistic function outputs and evaluate the performance of the family of classifiers defined by varying the threshold applied to this continuous output via the AUC of the ROC curve.

In these studies, standard parameters were selected without any adjustment to improve performance. We have found that generally the algorithm is not very sensitive to the choice of parameters. The following general considerations can be used to guide parameter selection.

- Number of training/test set realizations and proportion of samples used for training vs testing

The number of training/test set realizations was set at 325 or 375, with 2/3 of the samples used for training in most cases. The fraction of samples to use in training is chosen based on a trade-off between maintaining enough samples in training to represent the population adequately and providing diversity within the ensemble of training/test splits. Note that the considerations for this *p > N* setting with ensemble averaging are not precisely those normally considered in large datasets with *p < N* or those where cross-validation approaches are used. We have found that using 2/3 of samples in training works well in most cases, although it can be beneficial to increase the proportion of samples used in training when *N* is very small or there are reasons to believe that *N* is too small to represent the population. We chose to keep the ratio at 2/3 for these investigations, even though this may impact performance of the approach at the smallest sample sizes. With a training set: test set ratio of 2:1, generating 325 realizations ensures that on average each sample will be in the test set more than 100 times. Each time the sample is in the test set, we obtain an out-of-bag classification from a base classifier constructed based on a different associated training set. While it will never be possible to average over a meaningful fraction of the total number of possible training sets that can be generated holding a particular sample in the test set, sampling of 100 provides some sampling of diversity and convergence of the ensemble average.

b. kNN classification parameters

The kNN classifiers used a Euclidean distance and k of 7 or 9, as specified for each classification problem below. The optimal number of neighbors used in the nearest-neighbor algorithm depends on the particular classification problem, and in general will not be known for a specific real-world dataset. Often k is taken to be √*N* [25, 26], although some studies have suggested *N*^*x*^ with *x* between 0.25–0.375, depending on sample proportions and underlying covariance structure for small numbers of samples [27].

c. Filtering metric and range

In these settings of binary classification, the natural metric to assess the classification performance of atomic classifiers is accuracy. As the goal is only to discard atomic classifiers showing little or no indication of classification power, the range of the filter should be set wide, bearing in mind that the accuracy assessment is performed on the training set of the classifier and so will be over-optimistic. Filtering parameters were chosen so that around 25% of atomic classifiers passed filtering. We have found from previous experience that this is a reasonable choice in a variety of real world datasets. The performance of the classifier should be relatively stable over a variety of filtering widths as long as it is wide enough to incorporate a diversity of useful feature information and the regularization (see below) is strong enough.

d. Dropout parameters

Ten atomic classifiers were chosen for each dropout iteration and the number of dropout iterations was taken to be 100,000. The number of atomic classifiers selected in each dropout iteration, *d*, should be smaller than the number of samples in the training sets. The smaller *d* is, the greater the regularization. We have found from experience that *d* = 10 works in most settings where we have thousands of atomic classifiers passing filtering. In settings where far fewer atomic classifiers are to be combined, it is advisable to choose a smaller *d*. Once *d* has been selected, the number of dropout iterations should generally be selected to ensure that each atomic classifier passing filter should be sampled multiple times, typically 10–100. For all applications here, 100,000 dropout realizations are sufficient to reach this target. For the problem including many randomly generated features, this number is smaller than would be required to sample each atomic classifier multiple times, and some atomic classifiers may not be sampled at all for each master classifier. This can be viewed as an additional within bag random feature selection, as used in the standard RF.

#### Random Forest

The Random Forest was implemented as an ensemble average over trees, each constructed using the same training/test set realizations defined for the DRC approach. Hence, the training sets for each tree were subsets of the development set, drawn without resampling, stratified by class. This is advantageous, as it has been shown that use of sampling unstratified by class can produce unreliable out-of-bag estimators for the Random Forest in this setting of small sample size [28].

Again, standard (default) parameters were taken where possible and not adjusted to optimize performance [20]. The number of features randomly selected for each tree in the Random Forest was the square root of the number of samples, unless there were 30 or fewer samples per class (20 per class for training), in which case the number of features randomly selected for each tree was one third of the number of samples. An ensemble average over 325 trees and training:test ratio per tree of 2:1 was taken to match the training/test splits used for the DRC approach. To define an AUC to characterize classifier performance, a continuous classifier output was generated for each sample by averaging the class label (defined as 0 or 1) obtained for each tree over the ensemble.

#### kNN and logistic regression (single and bagged)

The individual and bagged kNN classifiers were constructed using Euclidean distance and the same k used as within the dropout-regularized combination approach (k = 7 or 9). Bagged kNN and bagged logistic regression were carried out using the same training/test set realizations as used for the other classification approaches. To define an AUC for the individual kNN approach, the kNN algorithm was extended to produce a score, defined as the number of neighbors in class 1.

### Classifier performance assessment

Our aim was to assess the relative performance of the families of binary classifiers generated by the methods under comparison. We did not want to compare one binary classifier optimized by tuning parameters or threshold for one method with another optimized for a different method. This was in line with our choices of standard parameters, fixed for each application.

Hence, performance of each family of classifiers was assessed via area under the ROC curve. This metric was considered most appropriate as we were interested in the rankings of the samples according to the continuous classifier output [20, 29].

Performance was evaluated for bagged approaches by out-of-bag estimates within the development set. Further, the AUC was evaluated via internal validation on any part of the development set not used for classifier generation and additionally on the full development data set with a combination of out-of-bag estimators for samples used in classifier generation and standard application of the classifier for other samples. Finally, the AUC was also determined for each classifier for an independent validation set.

### Datasets and details of classifier development

The datasets used in this work were selected from the GEO database as suitable for classifier development tasks in the precision medicine setting. These mRNA expression datasets are publically available at http://www.ncbi.nlm.nih.gov/geo. We required the datasets to have sufficient associated clinical data to formulate a meaningful classification task and the existence of two independent sets so that one could be used for development and the other set aside for independent validation.

#### Testing the ability of the classifier development method to work well with small datasets: predicting ten year survival for patients with prostate cancer

Two datasets were identified that were sufficiently large to allow systematic investigation, had enough overlap of available features (genes) and compatible clinical data. Dataset GSE16560 was selected for development and GSE10645 for validation. The GSE16560 cohort is a subset of a population-based Swedish Watchful Waiting cohort of patients with localized prostate cancer [30]. The GSE10645 cohort was drawn from subjects from the Mayo Radical Retropubic Prostatectomy Registry [31]. Genomic data were collected for 281 subjects with the human 6 k transcriptionally informative gene panel for DASL (GPL5474) for GSE16560 and for 596 subjects with the DASL human cancer panel (GPL5858) for GSE10645. To allow for comparison of results between the two datasets, only the 343 genes common to both datasets were considered. Where multiple probes were used to assess a single gene, these were averaged. ComBat, a tool to adjust for batch effects in microarray expression data using empirical Bayes methods [32] (available at http://www.bu.edu/jlab/wp-assets/ComBat/Abstract.html) was used to render the two datasets comparable. Survival data were available for both cohorts and these were dichotomized by considering survival at 10 years. Dropping out subjects with insufficient follow up for this endpoint left 261 subjects (116 alive at 10 years) for development and 445 (342 alive at 10 years) for validation.

For the dropout regularized combination, the kNN atomic classifiers used k = 7 and all 343 single features and all 58,653 distinct pairs of features. Note that choice of k to suit such a wide range of sample sizes is difficult and we chose not to optimize the method on this parameter. k = 7 was the largest k that could be used for the smallest sample sizes studied. Atomic classifiers were filtered according to classification accuracy on the training set. Typically around 25% of the atomic classifiers passed filtering for each training/test split. 100,000 dropout iterations were averaged.

To investigate classifier performance as a function of development set size, classifiers were constructed using 9 realizations of subsets of sizes 210, 186, 168, 144, 120, 86, 72, 60, 48, 36, 30, 24, and 18 drawn from the development dataset (with equal numbers, *N*_*class*_, of samples in each class (alive at 10 years and dead at 10 years)). All parameters were maintained as specified above. For each classifier the AUC was evaluated on the development subset and on the independent validation set, and each was averaged over the 9 development subset realizations.

Single and bagged kNN classification used k = 7, to match k used in the DRC approach. As standard kNN and logistic regression are known to perform poorly when large numbers of features are used [5], each of these methods (individual and bagged) was investigated using all 343 features (genes) and smaller subsets of features selected according to t-test *p* value for univariate differential expression between patients alive at 10 years and those dying before 10 years.

#### Testing the ability of the classifier development method to work well for a dataset with very many, but few useful, features: predicting ten year survival for patients with prostate cancer

This investigation used the same datasets as above with the same goal to predict 10-year survival. Here we compared the DRC classifier approach with the RF. To mimic the situation of very many features, with only a few with utility for the problem in question, we added 10,000 randomly generated gaussian features (mean = 0 and standard deviation = 1) to both the development and validation data sets. For the DRC approach, rank-based kNNs were used as atomic classifiers, to avoid any problems with differences in scale between the original and randomly generated features. All kNN classifiers (k = 7) using the 10,343 features singly and pairs of features that passed single feature filtering were considered. Filtering was set as in the previous problem and resulted in around 25% of atomic classifiers considered passing filtering and 100,000 dropout iterations were used.

DRC and RF were generated using identical training/test set realizations for 9 subsets each of the development set with *N* = 24, 48, 60, 72, 84, 93, and 105 samples per class. All other parameters used were the same as listed above.

#### Testing the ability of the classifier development method to deal with confounding effects: four year survival for NSCLC

The dataset GSE50081 was used for development and GSE42127 for validation. For the GSE50081 cohort expression profiling was performed on RNA from frozen, resected tumor tissue from 181 subjects with stage I or II NSCLC using Affymetrix Human Genome U133 Plus 2.0 Array (GPL570) [33]. Two patients with adenosquamous histology tumors were excluded from our studies. Expression profiling for the GSE42127 cohort was performed for 176 subjects with stage I-IV NSCLC on frozen tissue using the Illumina Human WG-6 v3.0 expression beadchip (GPL6884) [34]. Thirty-two patients with disease stage III, IV or unknown were not included in the analysis. Only the 15,005 genes in common between the two datasets were used, measurements were averaged over multiple probes for single genes where relevant, and the datasets were rendered comparable using ComBat. Both cohorts had survival data available and these were dichotomized by considering survival at 4 years. Dropping out subjects with insufficient follow up for this endpoint left 158 subjects (105 alive at 4 years (28 squamous and 77 non-squamous) and 53 dead (12 squamous and 41 non-squamous) at 4 years) for development and 105 (72 alive (16 squamous and 56 non-squamous) and 33 dead (9 squamous and 24 non-squamous) at 4 years) for validation.

For this problem, the DRC method used kNN (k = 9) atomic classifiers containing only single features, filtered by classification accuracy (alive or not at 4 years) on the training set, with 10 atomic classifiers randomly selected from the filtered pool for each of the 100,000 dropout iterations. The 9 subjects with non-squamous histology whose four-year survival status could not be unambiguously determined due to inadequate follow up were used as an additional sample set during filtering. We demanded that not all of these non-squamous subjects be classified as dead before 4 years, in addition to requiring sufficient classification accuracy for the training set. The resulting classifier was tested on the validation set as well as in the 9 subjects with non-squamous histology who could not be unequivocally classified as surviving at least 4 years or less than 4 years.

#### Software

Software implementing the methods presented in this study is available at https://bitbucket.org/diagnosticcortex/biodesixdxcortex1.

## Additional file

Additional file 1 **Table S1.** Classifier Development Parameters: Prognosis of Prostate Cancer Patients. **Table S2.** Classifier Development Parameters: Prognosis of Prostate Cancer Patients with 10,000 additional randomly generated features. **Table S3.** Classifier Development Parameters: Prognosis of Lung Cancer Patients After Surgery (DOCX 20 kb)

## Data Availability

The datasets supporting the conclusions of this article are in the Gene Expression Omnibus under GSE16560, GSE10645, GSE50081, and GSE42127. Datasets as preprocessed prior to classifier development are available in the BiodesixDxCortex1 repository at https://bitbucket.org/diagnosticcortex/biodesixdxcortex1/FullData. Software implementing the method presented in this study is available at https://bitbucket.org/diagnosticcortex/biodesixdxcortex1. Software requirements include: ● Operating System – Developed on Windows Server 2012 R2 Standard ● Programming Languages – Matlab R2017a, C# with. Net 4.5 ● Third Party Required Software - Matlab R2017a, Roguewave IMSL 6.5.0 ● Other Requirements – Example data is provided in the repository ● License – New (3-clause) BSD license

## Abbreviations

AUC: Area under the curve
Dev: Development
DRC: Dropout-regularized combination
GEO: Gene Expression Omnibus
Int Val: Internal validation
kNN: k-nearest neighbour
mRNA: Messenger Ribonucleic Acid
NSCLC: Non-small cell lung cancer
OOB: Out-of-bag
RF: Random Forest
ROC: Receiver-operating characteristic
SE: Standard error
Val: Independent validation
