# Supplementary material for: A dropout-regularized classifier development approach optimized for precision medicine test discovery from omics data
Source: BMC Bioinformatics. 2019 Jun 13;20:325. doi: 10.1186/s12859-019-2922-2 (PMC6567499; doi:10.1186/s12859-019-2922-2)
Supplement: Supplementary file 1 — Table S1. Classifier Development Parameters: Prognosis of Prostate Cancer Patients. Table S2. Classifier Development Parameters: Prognosis of Prostate Cancer Patients with 10,000 additional randomly generated features. Table S3. Classifier Development Parameters: Prognosis of Lung Cancer Patients After Surgery (DOCX 20 kb) [file 12859_2019_2922_MOESM1_ESM.docx]

**Additional file**

**Additional file 1: Table S1: Parameters used in classifier development: Prognosis of Prostate Cancer Patients**

| Method | Parameter | Value(s) |
| --- | --- | --- |
| DRC | k | 7 |
|  | Subsets of features used in the atomic classifiers | Singles and pairs |
|  | Atomic classifier filtering criteria | Overall accuracy |
|  | Atomic classifier filtering range | [0.68; 0.98] |
|  | Number of dropout iterations | 100,000 |
|  | Number of atomic classifiers kept in each dropout iteration | 10 |
|  | Number of training / test realizations | 325 |
|  | Training proportion in each realization | 2/3 |
| Single kNN classifier | k | 7 |
| Bagged kNN classifier | k | 7 |
|  | Number of training / test realizations | 325 |
|  | Training proportion in each realization | 2/3 |
| Bagged logistic regression classifier | Number of training / test realizations | 325 |
|  | Training proportion in each realization | 2/3 for both Alive and Dead classes |
| RF | Number of training / test realizations = trees | 325 |
|  | Training proportion in each realization | 2/3 for both Alive and Dead classes |
|  | Number of features considered at each node | $\sqrt{\# samples}$ (for # samples per class >30), (# samples)/3 otherwise |

**Additional file 1: Table S2: Parameters used in classifier development: Prognosis of Prostate Cancer Patients with 10,000 additional randomly generated features**

| Method | Parameter | Value(s) |
| --- | --- | --- |
| DRC | k | 7 |
|  | Subsets of features used in the atomic classifiers | Singles and pairs of features which pass single feature filtering |
|  | Atomic classifier filtering criteria | Overall accuracy |
|  | Atomic classifier filtering range | [0.68; 0.98] |
|  | Number of dropout iterations | 100,000 |
|  | Number of atomic classifiers kept in each dropout iteration | 10 |
|  | Number of training / test realizations | 325 |
|  | Training proportion in each realization | 2/3 |
| RF | Number of training / test realizations = trees | 325 |
|  | Training proportion in each realization | 2/3 for both Alive and Dead classes |
|  | Number of features considered at each node | $\sqrt{\# samples}$ (for # samples per class >30), (# samples)/3 otherwise |

**Additional file 1: Table S3: Parameters used in classifier development: Prognosis of Lung Cancer Patients After Surgery**

| Method | Parameter | Value(s) |
| --- | --- | --- |
| DRC | k | 9 |
|  | Subsets of features used in the atomic classifiers | Singles |
|  | Atomic classifier filtering criteria | Overall accuracy in the training set and overall accuracy in the additional set |
|  | Atomic classifier filtering range applied to the training set | [0.55; 1.0] |
|  | Atomic classifier filtering range applied to the additional set | [0.4; 1.0],  [0.5; 1.0],  [0.6; 1.0],  [0.7; 1.0],  [0.8; 1.0] and  [0.9; 1.0] |
|  | Number of dropout iterations | 100,000 |
|  | Number of atomic classifiers kept in each dropout iteration | 10 |
|  | Number of training / test realizations | 375 |
|  | Training proportion in each realization | 24/35 for the Alive class and 24/53 for the Dead class |
